# Hyperactive neuronal autophagy depletes BDNF and impairs adult hippocampal neurogenesis in a corticosterone-induced mouse model of depression

**DOI:** 10.7150/thno.81067

**Published:** 2023-01-22

**Authors:** Kuo Zhang, Fan Wang, Mengying Zhai, Meiyao He, Yuxuan Hu, Lijin Feng, Yuting Li, Jingyu Yang, Chunfu Wu

**Affiliations:** Department of Pharmacology, Shenyang Pharmaceutical University, Shenyang, 110016, China.

**Keywords:** Corticosterone, Depression, BDNF, Adult hippocampal neurogenesis, Neuronal autophagy.

## Abstract

**Background:** Depression is a mental disorder that poses a serious threat to human health. Adult hippocampal neurogenesis (AHN) is closely associated with the efficacy of antidepressants. Chronic treatment with corticosterone (CORT), a well-validated pharmacological stressor, induces depressive-like behaviors and suppresses AHN in experimental animals. However, the possible mechanisms of chronic CORT action remain elusive.

**Methods:** A chronic CORT treatment (0.1 mg/mL, drinking water for 4 weeks) was applied to prepare a mouse model of depression. Immunofluorescence was performed to analyze the hippocampal neurogenesis lineage, and immunoblotting, immunofluorescence, electron microscopy, and adeno-associated virus (AAV) expressing a pH-sensitive tandemly tagged light chain 3 (LC3) protein were used to analyze neuronal autophagy. AAV-hSyn-miR30-shRNA was used to knock down autophagy-related gene 5 (Atg5) expression in the neurons.

**Results:** Chronic CORT induces depressive-like behaviors and decreases the expression of neuronal brain-derived neurotrophic factor (BDNF) in the dentate gyrus (DG) of the hippocampus in mice. Moreover, it markedly diminishes the proliferation of neural stem cells (NSCs), neural progenitor cells, and neuroblasts and impairs the survival and migration of newborn immature and mature neurons in the DG, which may be attributed to changes in the cell cycle kinetics and induction of NSCs apoptosis. Furthermore, chronic CORT induces hyperactive neuronal autophagy in the DG, possibly by increasing the expression of ATG5 and causing excess lysosomal degradation of BDNF in neurons. Notably, inhibiting hyperactive neuronal autophagy in the DG of mice by knocking down Atg5 in neurons using RNA interference reverses the decrease of neuronal BDNF expression, rescues AHN, and exerts antidepressant effects.

**Conclusion:** Our findings reveal a neuronal autophagy-dependent mechanism that links chronic CORT to reduced neuronal BDNF levels, AHN suppression and depressive-like behavior in mice. In addition, our results provide insights for treating depression by targeting neuronal autophagy in the DG of the hippocampus.

## Introduction

Depression is a mental disorder that endangers human health, and its incidence rate has been increasing dramatically 1. Although the pathogenesis underlying depression has not been clearly elucidated, biological, psychological, and socio-environmental factors are generally believed to be involved 2. Among the biological factors, stress-related hormones have attracted extensive attention. Patients with depression show increased serum cortisol levels 3, 4, and the long-term administration of glucocorticoids, such as corticosterone (CORT), induces depressive-like behaviors in experimental animals 5, 6, suggesting that stress-related hormones play an important role in depression.

Many studies have indicated that adult hippocampal neurogenesis (AHN) plays an important role in the mechanism of CORT-induced depression. AHN, which transforms neural stem cells (NSCs) into mature neurons in the subgranular zone (SGZ) of the hippocampal dentate gyrus (DG), is closely associated with depressive behavior and stress reactivity 7-9. Furthermore, CORT substantially reduces AHN in experimental animals 5, 10, 11, antidepressants can enhance AHN, while inhibiting AHN reduces or eliminates some of the antidepressant effects 7, 9, 11, 12. These observations suggest the close association of CORT-induced depression and AHN. However, the possible mechanisms of CORT on AHN have not been elucidated, especially the effect of CORT on the NSC lineage in AHN. Brain-derived neurotrophic factor (BDNF), one of the most important factors affecting AHN 13, can regulate cell proliferation, cell survival, and neuronal synapse formation. Increases in BDNF associated with either exercise or antidepressant use can enhance AHN 14. Although studies have reported that CORT reduces hippocampal BDNF levels 15, 16, the possible mechanisms underlying the ability of CORT to reduce BDNF are largely unknown.

Autophagy is a process by which cells degrade cytoplasmic proteins or organelles via the lysosomal degradation pathway, and it helps supply the cell with energy, maintain cellular homeostasis, and regulate cell survival and death 17. It has been shown that autophagy may mediate CORT-induced AHN damage 18. Stress/CORT-induced autophagic death of NSCs attenuates AHN and induces depressive-like behaviors and cognitive defects 18, suggesting that aberrant autophagy is a trigger for AHN attenuation and depressive-like behavior. In addition, chronic unpredictable mild stress may strongly enhance hippocampal autophagy, decrease hippocampal BDNF expression, and induce depressive behavior in experimental animals 19-21; however, the mechanism has not been clarified. Thus, further investigations are required to determine whether CORT affects BDNF and AHN by affecting autophagy and ultimately inducing depressive-like behaviors.

Here, we report that CORT induces hyperactive autophagy in DG neurons in mice by increasing ATG5 expression, which results in excess lysosomal degradation of BDNF in neurons. Subsequently, neuronal BDNF expression in the DG is significantly decreased, leading to NSCs depletion, aberrant AHN, and depressive-like behaviors. Conversely, inhibiting neuronal autophagy in the DG by knocking down Atg5 in neurons using RNA interference reverses downregulated neuronal BDNF expression, rescues AHN, and exerts antidepressant effects. Our findings reveal for the first time that CORT acts via a neuronal autophagy-dependent mechanism to reduce neuronal BDNF, suppress AHN and induce depressive-like behavior in mice. Our results will hopefully provide a potential strategy to treat depression by targeting neuronal autophagy in the hippocampal DG.

## Materials and Methods

### Animals

Adult 8-week-old male C57BL/6J mice (18-22 g) were purchased from Beijing Huafukang Biotechnology Co., Ltd. (Beijing, China; certificate number SCXK (Jing) 2019-0008). The mice were housed under standardized environmental conditions (12 h alternating light and dark, 23 ± 2 ℃) and given free access to food and water. The experimental protocol was approved by the Experimental Animal Research Committee of Shenyang Pharmaceutical University (Shenyang, China; SYPU-IACUC-C2021-4-15-115) and experiments were conducted in accordance with the relevant institutional and national guidelines and regulations. Every effort was made to minimize the number of animals used and alleviate their suffering.

### Protocol to establish CORT-induced depression

The CORT-induced depression model was established according to a previous study, with minor modifications 6. CORT (purity >98%, Tokyo Chemical Industry, Tokyo, Japan) was dissolved in anhydrous ethanol and mixed with drinking water to a final concentration of 0.1 mg/mL CORT and 1% ethanol. Mice were exposed to CORT in drinking water for 8 weeks. Mice in the control group were only exposed to 1% ethanol.

### Experimental design

The experimental design for behavioral testing is shown in Figure S1A. Behavioral tests were performed between the 7th and 8th weeks of the CORT protocol.

The experimental design for evaluating AHN is shown in Figure 2A and 4A. To evaluate NSCs, NPCs and neuroblasts, mice were sacrificed after 8 weeks of CORT administration, and the brains were excised for immunofluorescence staining. To evaluate newborn immature and mature neurons, the mice were injected with BrdU (75 mg/kg) intraperitoneally once per day for 7 days after 4 weeks of CORT administration. The mice were sacrificed after 8 weeks of CORT administration, and the brains were excised for immunofluorescence staining.

The experimental design for evaluating the cell cycle is shown in Figure 3A. After 8 weeks of CORT administration, mice were intraperitoneally injected with BrdU (150 mg/kg). Approximately 22 h later, the mice were sacrificed, and the brains were excised for immunofluorescence staining.

The experimental design for evaluating autophagy flux is shown in Figure 5H. After 4 weeks of CORT administration, the mice were injected with AAV-CMV-mCherry-EGFP-LC3 in the brain via stereotactic surgery. After 8 weeks of CORT administration, the mice were sacrificed, and the brains were excised for immunofluorescence staining.

The experimental design for evaluating Atg5 knockdown in DG neurons is shown in Figure 7Q. Mice were injected with AAV-hSyn-miR30-shRNA (Atg5) or AAV-hSyn-miR30-shRNA (scramble) in the brain via stereotactic surgery. After 4 weeks of CORT administration, the mice were intraperitoneally injected with BrdU (75 mg/kg/day) for 7 days. After 8 weeks of CORT administration, the mice were sacrificed, and the brains were excised for immunofluorescence staining.

### Behavioral tests

#### Body weight

Weight loss is an important indicator of depression. Thus, body weight was measured once per week for all the mice.

#### Spontaneous locomotor activity

Spontaneous locomotor activity was assessed as previously described, with some modifications 22. The mice were placed in a corner of the autonomic activity box and allowed to explore the area freely for 6 min. An infrared high definition (HD) camera was positioned directly above the box to track and record the distance covered and the speed of each mouse. All parameters were analyzed using EthoVision XT 8.0 software (Noldus, Wageningen, Netherlands). After each test, the box was cleaned with 75% ethanol to remove any residual odors.

### Novel object recognition test

The novel object recognition (NOR) test, which is used for assessing cognitive ability of animals, was divided into three sessions as previously described with some modifications 23. An infrared HD camera was used to record mouse movement. In the first phase, the mouse was exposed to an empty locomotor activity box (50 × 50 × 50 cm^3^) for 5 min. Then, two identical objects were placed in the locomotor activity box, and the mouse was allowed to explore freely for 5 min. During the last stage, one of the identical objects was replaced by a new object in the same location and the time spent exploring each object and the discrimination index were measured. The discrimination index was calculated as the ratio of the time spent on a new object to the sum of the time spent on the original and new objects. The apparatus was cleaned with 75% ethanol after each test to remove residual odors. The movement of mice was analyzed using EthoVision XT 8.0 software (Noldus).

### Elevated plus maze

The elevated plus maze (EPM) test was performed to assess anxiety-like behavior of mice, as previously described, with some modifications 12. The EPM was a plus-cross-shaped apparatus consisting of four arms, two open arms without walls (30 × 5 cm^2^), and two closed arms with walls (30 × 5 × 15 cm^3^). In the center of the cross was an open platform (5 × 5 cm^2^). The maze was 50 cm from the floor, and an infrared HD camera was placed above the apparatus to record the movement of each animal. Each mouse was placed in the central zone facing the closed arm and allowed to explore freely for 5 min. The total distance moved and time spent on each arm were recorded. The EPM test was performed in a dim room. The apparatus was cleaned with 75% ethanol after each test to remove residual odors. All parameters were analyzed using EthoVision XT 8.0 software (Noldus).

### Open field test

The open field test (OFT) was performed to investigate anxiety-like behavior, as previously described, with some modifications 24. Mice were placed individually in a corner of a box (50 × 50 × 30 cm^3^) in a dimly lit room. The mice were allowed to explore freely for 6 min. An infrared HD camera was positioned directly above the box to track and record the movement of each mouse. The total distance covered, speed, and time spent in the perimeter, non-perimeter, and central area were recorded using EthoVision XT 8.0 software (Noldus). The apparatus was cleaned with 75% ethanol after each test to remove residual odors.

### Tail suspension test

The tail suspension test (TST) was performed to assess depression-like behavior as previously described, with some modifications 25. Briefly, the mice were suspended approximately 1 cm from the tip of the tail and fixed using adhesive tape for 6 min. The immobility time was recorded during the final 4 min of the test and analyzed using EthoVision XT 8.0 software (Noldus).

### Forced swimming test

The forced swimming test (FST) was performed as previously reported, with some modifications 26. Mice were placed for 6 min in a transparent plexiglass cylinder (12 cm in diameter × 40 cm in height) containing water at a depth of 10 cm and 23 ± 1 °C. The immobility time was recorded during the final 4 min of the test and analyzed using EthoVision XT 8.0 software (Noldus).

### Confocal imaging and quantification

Confocal images of Z-series stacks were obtained (Nikon, Tokyo, Japan, Y-TV55) at 2048×2048 pixels, 1 × zoom, 0.125 scanning speed, and 10 μm (for 20 × objective) height. To quantify the total cell population in the DG of the bilateral hippocampus, brain slices (40 μm thick) containing the hippocampus were selected and stained. For all quantifications, pictures from at least six brain slices/mouse were acquired. The number of cells was counted using ImageJ software (National Institutes of Health, Bethesda, MD, USA). The volume of the GCL was determined by multiplying the surface area with the slice thickness. The density of positive cells was calculated by dividing the number of positive cells by the corresponding volume of GCL.

### Stereotactic surgery

The mice were anesthetized with isoflurane and placed on a stereotaxic apparatus (RWD Life Science, Shenzhen, China). AAV-CMV-mCherry-EGFP-LC3, AAV-hSyn-miR30-shRNA (Atg5), or AAV-hSyn-miR30-shRNA (scramble) was injected individually into both sides of the DG (1 μL/side) using a micro-syringe (Hamilton, Reno, NV, USA) and a microinjection pump (KD Scientific, Holliston, MA, USA) at a rate of 0.2 μL/min. Based on the mouse brain atlas of Pixinos, the coordinates for injection (total volume of 1 μL/side) were as follows: anterior posterior (AP), -1.00 mm; medial lateral (ML), ±1.94 mm; and dorsoventral (DV), -2.20 mm. The titers of AAV-CMV-mCherry-EGFP-LC3, AAV-hSyn-miR30-shRNA (Atg5), and AAV-hSyn-miR30-shRNA (scramble) were 1.5 × 10^12^ vg/mL, 1.9 × 10^12^ vg/mL, and 1.3 × 10^12^ vg/mL, respectively. All viruses were packaged at HanBio Biotechnology (Shanghai, China).

### Electron microscopy

After anesthetization with isoflurane, mice were perfused transcardially with 0.9% saline, followed by 4% paraformaldehyde (PFA) and 2.5% glutaraldehyde solutions. The brains were dissected, and the DG of the hippocampus was isolated immediately on an icebox. DG tissues were fixed in 2.5% glutaraldehyde at 4 °C and post-fixed in osmic acid at 25 ℃. Different concentrations of ethanol and acetone were used for gradient dehydration, and propylene oxide and resin were used for infiltration and tissue embedding. The ultrastructures of autophagosomes and autolysosomes were observed using transmission electron microscopy.

### Immunofluorescence

After deep anesthesia with isoflurane, mice were perfused with saline and 4% PFA. Brains were fixed in 4% PFA overnight, transferred to 0.4% PFA, and stored at 4 ℃. The brain tissues were sliced into 40 μm coronal sections (Leica, Wetzlar, Germany), and slices of the dorsal, middle, and ventral hippocampi were randomly selected for immunostaining. Sections were washed with phosphate buffer saline (PBS, 0.01 M) twice for 5 min each followed by antigen retrieval in citrate buffer at 80 ℃ for 30 min. For BrdU immunostaining, the slices were exposed to HCl (2 M) for 20 min at 37 ℃ and then washed in PBS (0.01 M) for 5 min and PBS (0.01 M) with 0.2% Triton X-100 (PBST) twice for 5 min each. Next, the slices were blocked with 5% pig serum in PBST for 1 h at 25 ℃. Subsequently, the sections were incubated with primary antibodies overnight at 4 ℃ and then incubated in the dark with fluorescently labeled secondary antibodies for 1 h at 25 ℃. Finally, the brain slices were stained with DAPI (Beyotime Biotechnology, Shanghai, China, C1006) for 20 min and washed twice. The primary antibodies used were chicken anti-GFAP (1:1500, Abcam, Cambridge, UK, ab4674), rabbit anti-SOX2 (1:1000, Abcam, ab97959), rabbit anti-DCX (1:500, Abcam, ab207175), rabbit anti-Tbr2 (1:1000, Abcam, ab223345), mouse anti-MCM2 (1:1000, BD, Franklin Lakes, NJ, USA, BM28), rat anti-BrdU (1:500, Abcam, ab6326), rabbit anti-BDNF (1:500, Abcam, ab213323), rat anti-LAMP2 (1:500, Abcam, ab13524), rabbit anti-cleaved caspase-3 (1:500, Cell signaling, 9664T), and mouse anti-NeuN (1:1000, Millipore, Bedford, MA, USA, MAB377). The secondary antibodies used were goat anti-chicken Alexa Fluor 647 (1:400; Abcam, ab150171), donkey anti-rabbit Alexa Fluor 555 (1:400; Abcam, ab150074), donkey anti-rat Alexa Fluor 488 (1:400; Abcam, ab150149), donkey anti-mouse Alexa Fluor 488 (1:400; Abcam, ab150105), and donkey anti-mouse Alexa Fluor 647 (1:400; Abcam, ab150107).

### Western blot analysis

Hippocampal DG tissues were homogenized in RIPA lysis buffer (Beyotime, P0013b) with proteinase inhibitors (Solarbio, Beijing, China) and phosphatase inhibitors (Solarbio, P1260). Then, the samples were centrifuged at 12,000 × *g* for 15 min at 4 °C. The supernatants or resuspended pellets were mixed with 5× sodium dodecyl sulfate (SDS) loading buffer (Solarbio, P1040). Before heating at 100 ℃ for 10 min, the protein concentrations were determined using a bicinchoninic acid kit (Beyotime, P0012). The proteins were separated on 10%-12% SDS polyacrylamide gels and transferred to polyvinylidene fluoride membranes (Millipore, IPVH00010). The membranes were blocked with 5% skimmed milk (BD Biosciences, San Jose, CA, USA) at 25 ℃ for 1 h and incubated with primary antibodies against LC3 (1:1000; MBL, PM036), SQSTM1/p62 (1:1000; Abcam, ab56416), Atg5 (1:1000; Abcam, ab108327), ULK1 (1:1000; Cell signaling, 8054), BDNF (1:1000; Abcam, ab108319) and β-actin (1:1000; Elabscience, Wuhan, China, E-AB-40338) overnight at 4 ℃. After washing three times with Tris-buffered saline containing Tween (TBST) for 5 min each, the membranes were incubated with the corresponding horseradish peroxidase-conjugated secondary antibody at 25 ℃ for 1 h. The membranes were washed three times with TBST for 5 min each, and immunoreactivity was detected using an enhanced chemiluminescence reagent kit (NCM Biotech Co., Ltd, Newport, RI, USA, P10300). The bands were analyzed using ImageJ software (National Institutes of Health).

### Quantitative real-time polymerase chain reaction (RT-PCR)

Total RNA was extracted from hippocampal DG tissues using TRIzol reagent (15596026, Invitrogen) according to the manufacturer's instructions. Then, the purified total RNA was reversely transcripted to cDNA using the PrimeScript^TM^ RT reagent kit with gDNA Eraser (Takara, Japan, RR047A). The qPCR assay was carried out using TB Green^®^ Premix Ex Taq™ II (Takara, Japan, RR820A). The amplification parameters were as follows: denaturation at 95°C for 30 s, annealing at 95°C for 5 s and extension at 60°C for 30 s for 40 cycles and the signal was detected at 60°C. Data were quantified using the 2^-△△Ct^ method and normalized to β-actin expression. Primer sequences (Sangon Biotech) were: *Bdnf*, 5'-CACTGGCTGACACTTTTGAGCAC-3' and 5'-GCTGTGACCCACTCGCTAATACTG-3'; *β-actin*, 5'-GGCTGTATTCCCCTCCATCG-3' and 5'-CCAGTTGGTAACAATGCCATGT-3'.

### Statistical analyses

All data are presented as the mean ± standard error of mean. Statistical analyses were performed using SPSS 21.0 software (IBM, Armonk, NY, USA), and two-tailed unpaired *t*-tests were performed to determine whether differences between the results of the two groups were statistically significant. Results at *P* < 0.05 were considered statistically significant, and non-statistical difference are indicated as n.s.

## Results

### CORT induces depressive-like behaviors and decreases neuronal BDNF expression in mice

**Figure 1:**
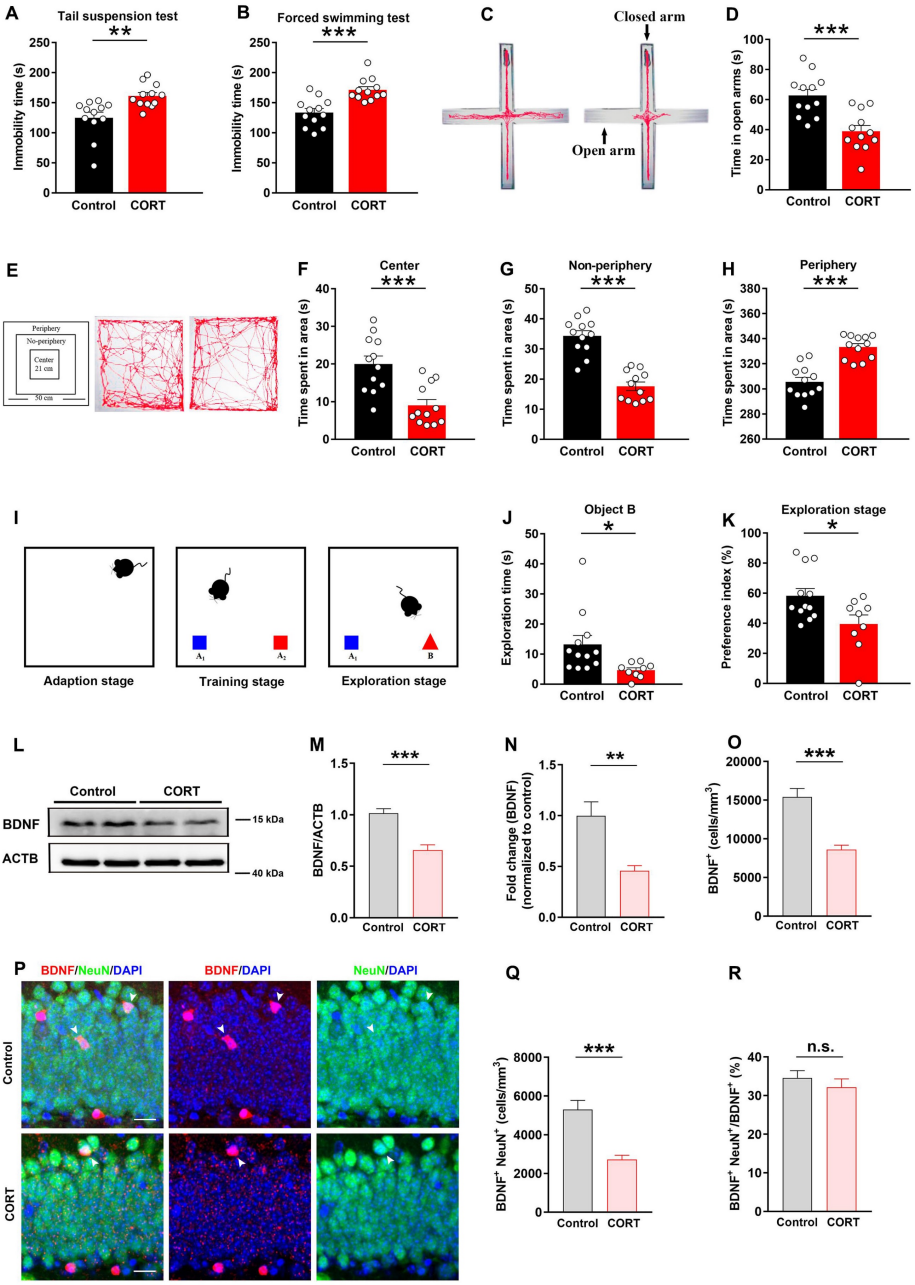
** CORT induces depressive-like behaviors, hippocampal-dependent cognitive deficits, reduced neuronal BDNF levels. (A)** Immobility time in the TST. n = 12 per group, *P* = 0.0029. **(B)** Immobility time in the FST. n = 12 per group, *P* = 0.0002. **(C)** Representative tracking plots of the EPM. **(D)** Time spent in the open arms of the EPM. n = 12 per group, *P* = 0.0003. **(E)** Representative tracking plots of the OFT. **(F)** Average time spent in the central area of the OFT. n = 12 per group, *P* = 0.0004. **(G)** Time spent in the non-periphery area of the OFT. n = 12 per group, *P* < 0.0001. **(H)** Time spent in the periphery area of the OFT. n = 12 per group, *P* < 0.0001. **(I)** Diagram of the NOR test.** (J)** Exploration time for the novel object B in the exploration stage of the NOR test. Control (n = 12), CORT (n = 9), *P* = 0.0237. **(K)** Preference index in the exploration stage of the NOR test. Control (n = 12), CORT (n = 9), *P* = 0.0248. **(L)** Representative western blots analyzing BDNF protein expression in the control and CORT groups.** (M)** Quantification of BDNF expression. n = 3 mice per group, *P* < 0.0001.** (N)** Quantification of BDNF gene mRNA expression. n = 4 mice per group, *P* = 0.0024.** (O)** Quantification of BDNF^+^ cells. n = 3 mice per group, *P* < 0.0001. **(P)** Representative images of the control and CORT DG with double immunostaining of BDNF^+^ (red) and NeuN^+^ (green) cells. Arrowheads indicate BDNF^+^/NeuN^+^ cells. **(Q)** Quantification of BDNF^+^NeuN^+^ cells. n = 3 mice per group, *P* < 0.0001. **(R)** Quantification of BDNF^+^NeuN^+^ cells/total BDNF^+^ cells. n = 3 mice per group, *P* = 0.4026. Scale bar = 20 μm. Data are presented as the mean ± standard error of mean (SEM). Two-tailed unpaired *t-*test was used to identify statistically significant differences between datasets (*P < 0.05, **P < 0.01, ***P < 0.001 compared to the control group). n.s., non-significant difference.

To determine whether CORT induces depressive-like behaviors in mice, we carried out the following tests: TST, FST, EPM and OFT. Compared to the control mice, CORT-treated mice exhibited increased durations of immobility in the TST (Figure 1A) and FST (Figure 1B). In the EPM test, CORT-treated mice spent less time exploring the open arms and more time exploring in the closed arms (Figure 1C, 1D and S1E). In the OFT, the CORT group spent less time in the central/non-peripheral area (Figure 1E, 1F and 1G) and more time in the peripheral area (Figure 1H). These results indicate that CORT induces depressive behavioral phenotypes in mice. As cognitive dysfunction is another common feature of depression, we subjected CORT and control mice to the NOR test, which assesses hippocampus-dependent cognitive ability. The data showed that CORT-treated mice presented notable cognitive deficits compared to the control mice, including decreased exploration time (Figure 1I and 1J) and preference index (Figure 1K) for novel objects, although differences were not observed in terms of the training stage (Figure S1G, S1H and S1I). Slow body weight gain is a characteristic feature of prolonged CORT administration. During the first week of the experiment, the body weight gain of the CORT-treated mice was significantly lower than that of the control mice (Figure S1B and S1C). Differences in the distance moved were not observed between the CORT-treated mice and control mice (Figure S1D and S1F), suggesting that CORT had no effect on mice in terms of locomotor activity. Thus, CORT induced several depression-related behavioral phenotypes and hippocampal-dependent cognitive deficits.

Western blot and immunofluorescence analysis revealed that the expression of BDNF in the hippocampal DG was significantly reduced in CORT mice compared to that in control mice (Figure 1L, 1M and 1O). Moreover, the mRNA expression of BDNF gene in the hippocampal DG was significantly reduced in CORT mice compared to that in control mice (Figure 1N). Significant reductions of BDNF were observed in the neurons of CORT mice (Figure 1P and 1Q), although obvious differences in the ratio of BDNF^+^NeuN^+^/BDNF^+^ cells were not observed in CORT and control mice (Figure 1R). These results suggest that the decreased expression of BDNF induced by CORT may be due to impaired synthesis of BDNF in DG neurons.

### CORT impairs the survival and proliferation of NSCs, neural progenitor cells (NPCs), and neuroblasts in mice

**Figure 2:**
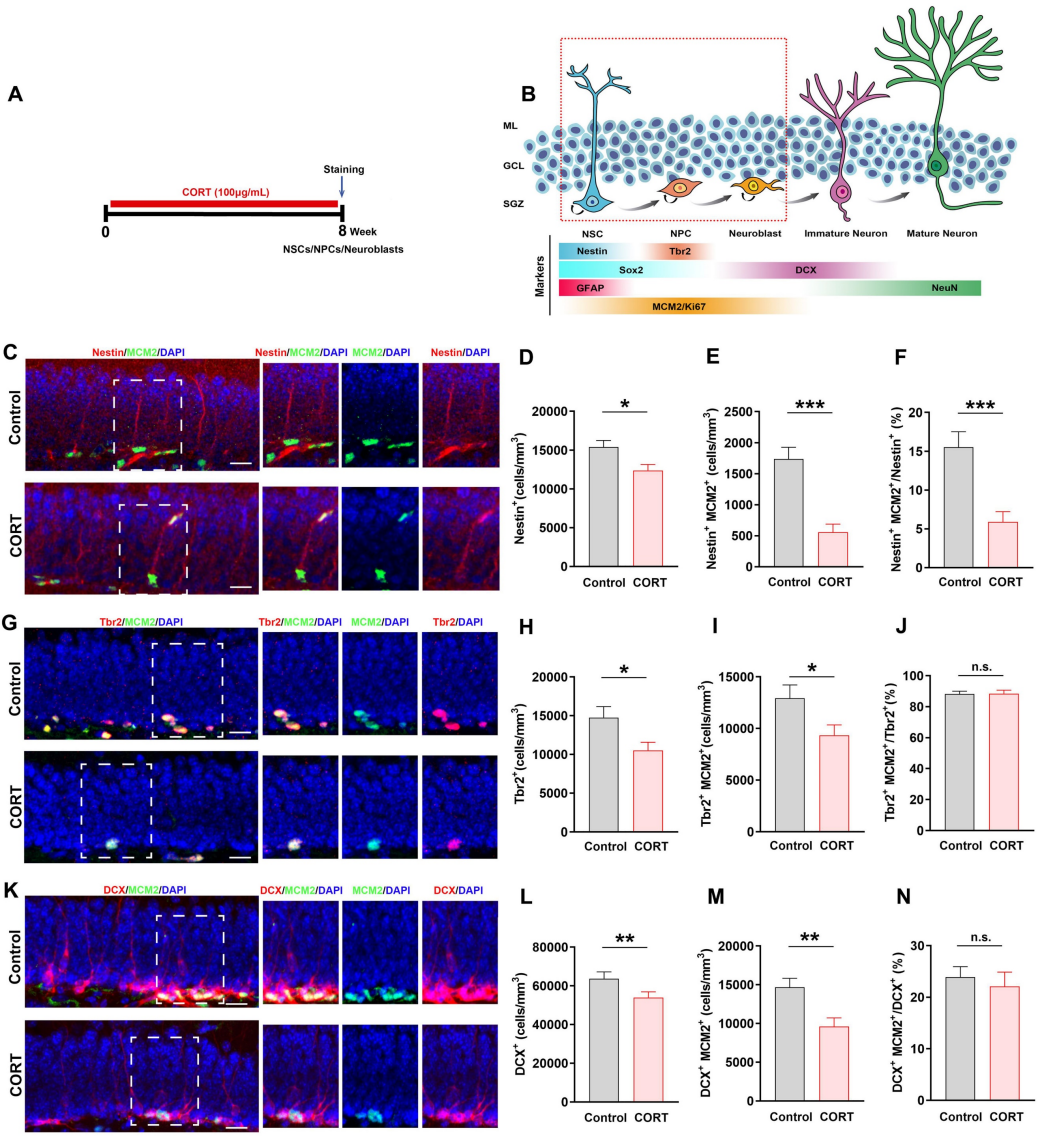
** Chronic CORT treatment depletes neural stem cells (NSCs), neural progenitor cells (NPCs), and neuroblasts, and impacts the proliferation of neural progenitor cells NSCs, NPCs, and neuroblasts. (A)** Timeline of the experimental procedure for assessing adult hippocampal neurogenesis (AHN). **(B)** Diagram showing the AHN lineage and markers. **(C)** Representative images of the control and CORT DG with double immunostaining of Nestin^+^ (red) and MCM2^+^ (green) cells. **(D)** Quantification of Nestin^+^ cells. n = 4 mice per group, *P* = 0.0095. **(E)** Quantification of Nestin^+^MCM2^+^ cells. n = 4 mice per group, *P* < 0.0001. **(F)** Quantification of Nestin^+^MCM2^+^/Nestin^+^ cells. n = 3 mice per group, *P* = 0.0006. **(G)** Representative images of the control and CORT DG with double immunostaining of Tbr2^+^ (red) and MCM2^+^ (green) cells. **(H)** Quantification of Tbr2^+^ cells. n = 4 mice per group, *P* = 0.0200. **(I)** Quantification of Tbr2^+^MCM2^+^ cells. n = 4 mice per group, *P* = 0.0309. **(J)** Quantification of Tbr2^+^MCM2^+^/Tbr2^+^ cells. n = 4 mice per group, *P* = 0.9473. **(K)** Representative images of the control and CORT DG with double immunostaining of DCX^+^ (red) and MCM2^+^ (green) cells. **(L)** Quantification of DCX^+^ cells. n = 4 per group, *P* = 0.0436. **(M)** Quantification of DCX^+^MCM2^+^ cells. n = 4 per group, *P* = 0.0033. **(N)** Quantification of DCX^+^MCM2^+^/DCX^+^ cells. n = 3 mice per group, *P* = 0.6096. Scale bar = 20 μm. Data are presented as the mean ± SEM. Two-tailed unpaired *t-*test was used to identify statistically significant differences between datasets (*P < 0.05, **P < 0.01, ***P < 0.001 compared to the control group). n.s., non-significant difference.

Based on the vital role of AHN in depression and cognition, we analyzed the developmental stage of cells undergoing AHN in CORT-treated mice (Figure 2A). As shown in Figure 2B, NSCs, neural progenitor cells (NPCs), neuroblasts, and proliferating cells are marked by Nestin, Tbr2, DCX, and MCM2 expression, respectively 13. The numbers of Nestin^+^ cells (Figure 2C and 2D) and Nestin^+^MCM2^+^ cells (Figure 2C and 2E) were considerably decreased in CORT mice compared to those in control mice, which suggests that the survival and proliferation of NSCs were impaired by CORT. Since NPCs and neuroblasts mainly originate from NSCs, damage to NSCs would affect NPCs and neuroblasts. As expected, the numbers of Tbr2^+^ (Figure 2G and 2H), Tbr2^+^MCM2^+^ (Figure 2G and 2I), DCX^+^ (Figure 2K and 2L), and DCX^+^MCM2^+^ cells (Figure 2K and 2M) were markedly decreased in CORT mice. Accordingly, the ratio of Nestin^+^MCM2^+^ cells/Nestin^+^ cells was substantially decreased (Figure 2F) in CORT mice. Interestingly, no obvious difference was observed in the ratios of Tbr2^+^MCM2^+^/Tbr2^+^ cells (Figure 2J) and DCX^+^MCM2^+^/DCX^+^ cells (Figure 2N) in CORT and control mice. These results indicate that CORT not only depleted NSCs, NPCs, and neuroblasts, but also affected the proliferation of NSCs, NPCs, and neuroblasts. Moreover, CORT significantly decreased the proportion of proliferating NSCs without influencing the proliferation of NPCs and neuroblasts. This suggests that the abnormal changes in NSCs may account for the CORT-induced impairment of their survival and proliferation.

### CORT impairs the cell cycle kinetics of NSCs and accelerates the apoptosis of NSCs in mice

**Figure 3:**
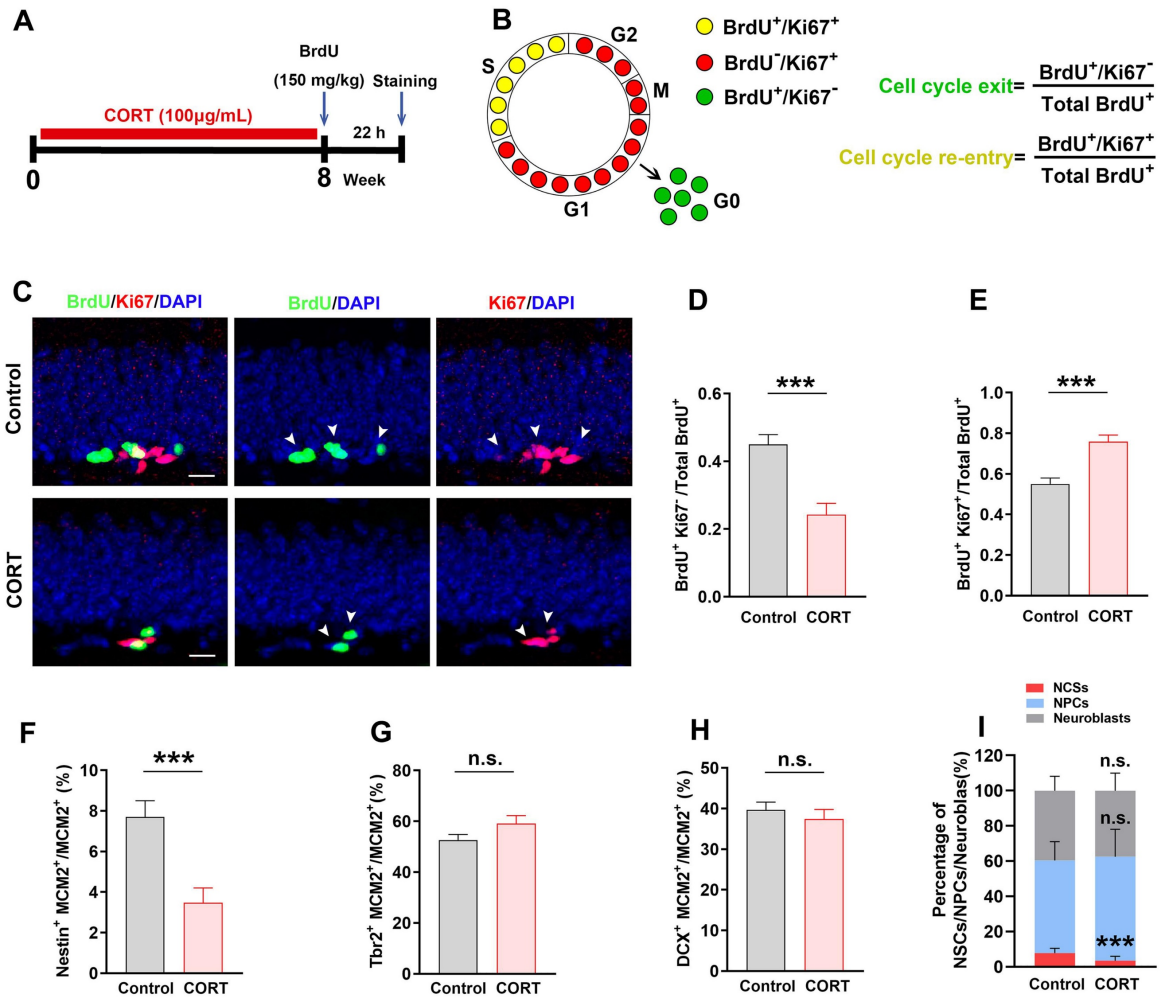
** Chronic CORT treatment impairs the cell cycle kinetics of NSCs. (A)** Timeline of the experimental procedure, including BrdU administration. **(B)** Schematic diagram showing how BrdU and Ki67 co-labeling reflects the different stages of the cell cycle. The equations for calculating the index of cell cycle exit and re-entry are shown at the right. **(C)** Representative images of the control and CORT DG with double immunostaining of BrdU^+^ (green) and Ki67^+^ (red). White arrowheads indicate Ki67^+^/BrdU^+^ cells. **(D)** Quantification of BrdU^+^Ki67^-^/total BrdU^+^ cells. n = 7 mice per group, *P* < 0.0001. **(E)** Quantification of BrdU^+^Ki67^+^/total BrdU^+^ cells. n = 7 mice per group, *P* < 0.0001. **(F)** Quantification of Nestin^+^MCM2^+^/MCM2^+^ cells. n = 3 mice per group, *P* = 0.0006. **(G)** Quantification of Tbr2^+^MCM2^+^/MCM2^+^ cells. n = 4 mice per group, *P* = 0.9473. **(H)** Quantification of DCX^+^MCM2^+^/MCM2^+^ cells. n = 3 mice per group, *P* = 0.2207. **(I)** The proportion of NSCs, NPCs, and neuroblasts among all proliferating cells (NSCs + NPCs + neuroblasts) in the DG of CORT and control mice. n = 3 mice per group, *P* = 0.0007. Scale bar = 20 μm. Data are presented as the mean ± SEM. Two-tailed unpaired *t-*test was used to identify statistically significant differences between datasets (*P < 0.05, ***P < 0.001 compared to the control group). n.s., non-significant difference.

After observing abnormal changes during the proliferative stage of AHN, we next assessed the cell cycle kinetics of proliferating cells. Bromodeoxyuridine (BrdU) and Ki67 were used to identify cell cycle exit and re-entry, respectively (Figure 3A). BrdU^+^Ki67^-^ cells were defined as cells that exited the cell cycle and BrdU^+^Ki67^+^ cells were defined as cells that re-entered the cell cycle (Figure 3B). Compared with control mice, the CORT mice showed a considerable reduction in the number of BrdU^+^Ki67^-^ cells (Figure 3C and 3D) and a considerable increase in the number of BrdU^+^Ki67^+^ cells (Figure 3C and 3E). This suggests that CORT promotes cell cycle re-entry. Next, we evaluated the proportion of NSCs, NPCs, and neuroblasts undergoing proliferation. The ratio of Nestin^+^MCM2^+^ cells/MCM2^+^ cells was substantially reduced (Figure 3F and 3I) in CORT mice compared to that in control mice. Interestingly, no obvious difference was observed in the ratios of Tbr2^+^MCM2^+^/MCM2^+^ cells (Figure 3G and 3I) and DCX^+^MCM2^+^/MCM2^+^ cells (Figure 3H and 3I) in CORT mice. These results indicate that CORT promoted cell cycle re-entry of NSCs and reduced the proportion of proliferating NSCs without affecting the proliferation of NPCs and neuroblasts. Since abnormal cell cycle changes were observed in NSCs, we investigated whether apoptosis was occurring in NSCs. The proportion of NSCs expressing cleaved-caspase 3 was significantly higher in CORT mice than in control mice (Figure S1J and S1K). These results indicate that CORT impairs the cell cycle kinetics of NSCs and accelerates the apoptosis of NSCs in mice.

### CORT impairs the survival of newborn immature/mature neurons and the migration of newborn mature neurons in the hippocampal DG of mice

**Figure 4:**
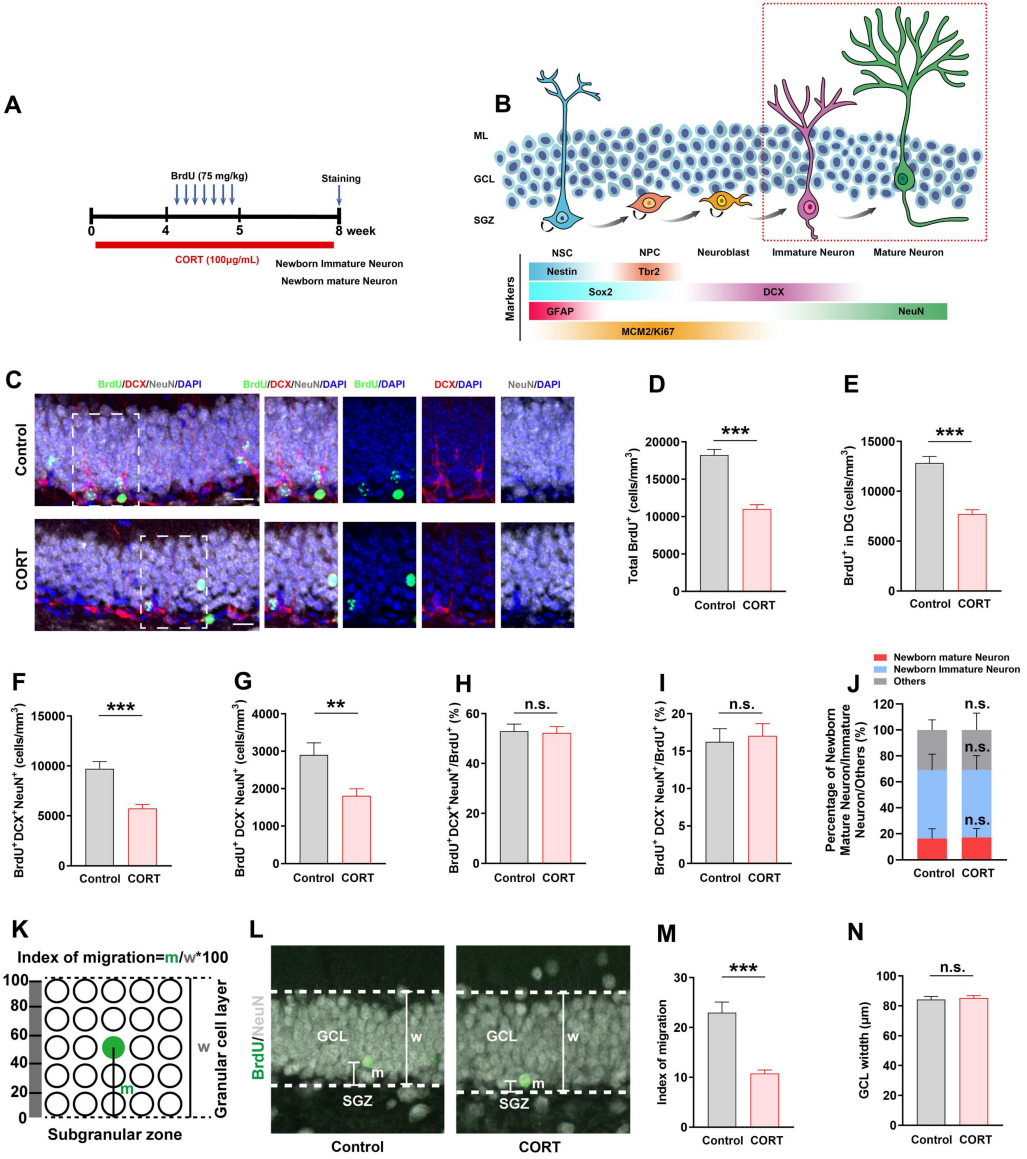
** Chronic CORT treatment impairs the survival of newborn immature/mature neurons and suppresses the migration of newborn mature neurons. (A)** Timeline of the experimental procedure. **(B)** Diagram of the AHN lineage and the markers used to identify the different cell types. **(C)** Representative images of the control and CORT DG with triple immunostaining of BrdU^+^ (green), DCX^+^ (red), and NeuN^+^ (gray) cells. **(D)** Quantification of total BrdU^+^ cells in the DG, hilus and molecular layer. n = 4 mice per group, *P* < 0.0001. **(E)** Quantification of BrdU^+^ cells in the DG. n = 4 mice per group, *P* < 0.0001. **(F)** Quantification of BrdU^+^DCX^+^NeuN^+^ cells. n = 4 mice per group, *P* < 0.0001. **(G)** Quantification of BrdU^+^DCX^-^NeuN^+^ cells. n = 4 mice per group, *P* < 0.0001. **(H)** Quantification of BrdU^+^DCX^+^NeuN^+^ cells/total BrdU^+^ cells. n = 4 mice per group, *P* = 0.8490. **(I)** Quantification of BrdU^+^DCX^-^NeuN^+^ cells/total BrdU^+^ cells. n = 4 mice per group, *P* = 0.7392. **(J)** Proportion of newborn mature neurons, newborn immature neurons, and other newborn cells among all newborn cells. n = 4 mice per group, *P* > 0.05. **(K)** Schematic representation of the calculation method used for assessing the migration of newborn neurons in GCL. **(L)** Representative images showing the migration of newborn mature neurons (BrdU^+^NeuN^+^) in GCL. **(M)** Quantification of the migration index in CORT and control mice. n = 4 mice per group, *P* < 0.0001. **(N)** Quantification of the GCL width. n = 4 mice per group, *P* = 0.7087. Scale bar = 20 μm. Data are presented as the mean ± SEM. Two-tailed unpaired *t-*test was used to identify statistically significant differences between datasets (**P < 0.01, ***P < 0.001 when compared to the control group). n.s., non-significant difference.

Four weeks after BrdU injection (Figure 4A), newborn immature and mature neurons were identified by their marker expression: DCX^+^NeuN^+^ for immature neurons and DCX^-^NeuN^+^ for mature neurons (Figure 4B). The data showed that the number of total BrdU^+^ cells in the DG, hilus and molecular layer (Figure 4C and 4D) and BrdU^+^ cells in the DG (Figure 4C and 4E) decreased substantially in CORT mice than that in control mice, which indicates that the number of newborn cells during AHN was greatly reduced. Next, we found that the number of newborn immature neurons (BrdU^+^DCX^+^NeuN^+^; Figure 4F) and newborn mature neurons (BrdU^+^DCX^-^NeuN^+^) (Figure 4G) was substantially reduced in CORT mice, while the proportion of newborn immature neurons (Figure 4H and 4J) and newborn mature neurons (Figure 4I and 4J) in CORT mice did not change compared to that in control mice. This indicates that CORT had no effect on the proportion of newborn immature neurons and mature neurons in AHN. Newborn mature neurons migrate from the SGZ to the granular cell layer (GCL), and the distance covered influences the integration of newborn mature neurons into neural circuits 3, 13, 27. Therefore, we investigated the effects of CORT on the migration of mature newborn neurons (Figure 4K). The data showed that CORT did not change the GCL width (Figure 4L and 4N) but substantially reduced the distance covered by the newborn mature neurons compared to that in control mice (Figure 4L and 4M). Together, these results indicate that CORT not only impairs the survival of newborn immature and mature neurons but also suppresses the migration of newborn mature neurons in the hippocampal DG of mice.

### CORT induces hippocampal DG neuronal autophagy and lysosomal degradation of neuronal BDNF in mice

**Figure 5:**
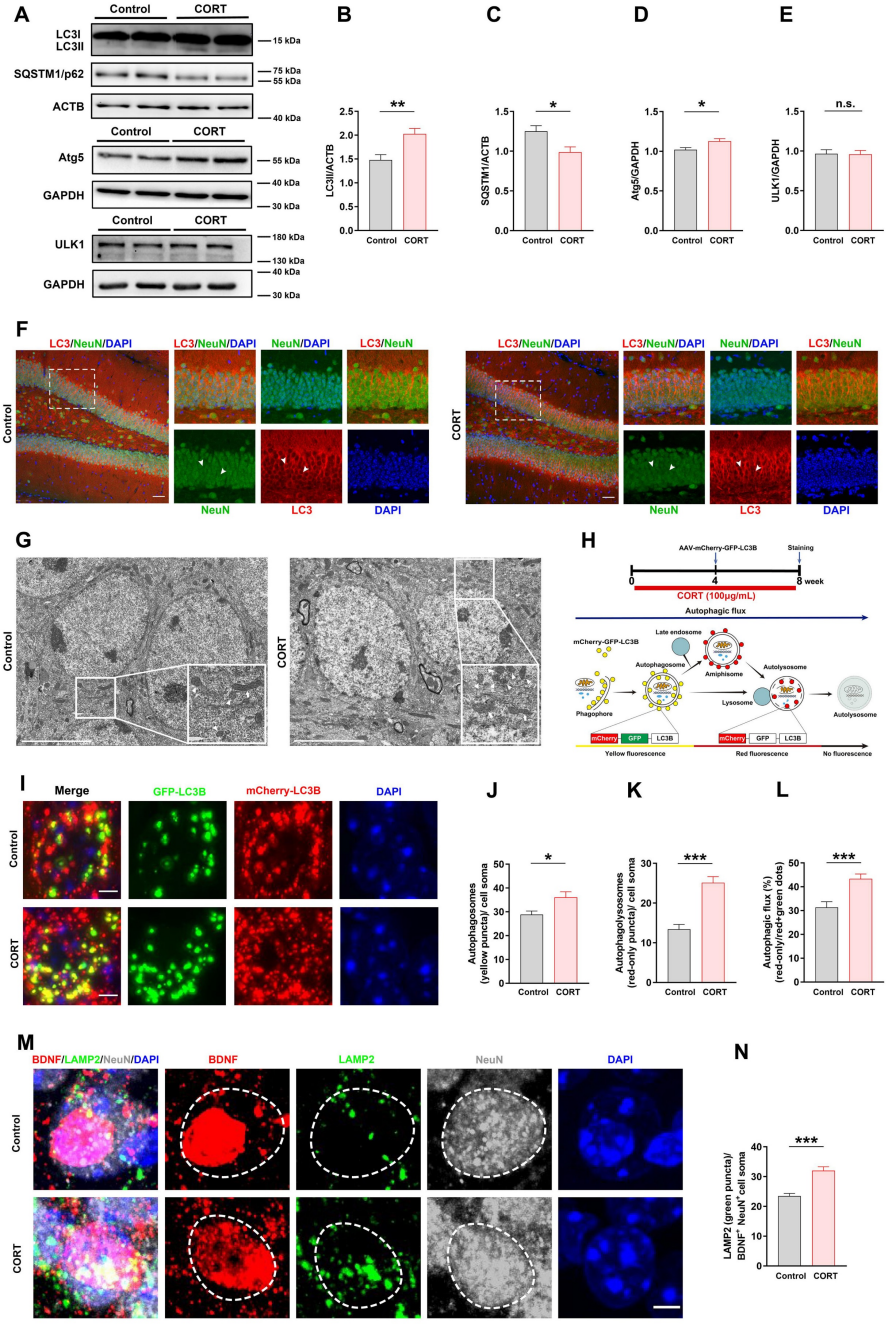
** Chronic CORT treatment induces hyperactive neuronal autophagy and promotes lysosomal degradation of neuronal BDNF in mice. (A)** Representative western blots analyzing protein expression in control and CORT groups. **(B)** Quantification of LC3II expression. n = 3 mice per group, *P* = 0.0015. **(C)** Quantification of SQSTM1/p62 expression. n = 3 mice per group, *P* = 0.0115. **(D)** Quantification of Atg5 expression. n = 3 mice per group, *P* = 0.0106. **(E)** Quantification of ULK1 expression. n = 3 mice per group, *P* = 0.9826. **(F)** Representative images of the control and CORT DG with double immunostaining of LC3^+^ (red) and NeuN^+^ (green) cells. Arrowheads indicate LC3^+^/NeuN^+^ cells. **(G)** Representative electron microscopic images of autophagosomes (white triangles) and autolysosomes (white arrows) in control and CORT mice. **(H)** Timeline of the procedure for the AAV-mCherry-GFP-LC3 experiment (top) and schematic diagram illustrating the detection of different autophagic structures by the mCherry-GFP-LC3 fusion protein (bottom). **(I)** Representative images of AAV-expressed mCherry-GFP-LC3 in control and CORT groups. **(J)** Quantification of autophagosomes (yellow puncta) in cell soma. n = 4 mice per group, *P* = 0.0111. **(K)** Quantification of autolysosomes (red-only puncta) in cell soma. n = 4 mice per group, *P* < 0.0001. **(L)** Quantification of autophagic flux. n = 4 mice per group, *P* = 0.0002. **(M)** Representative images of the control and CORT DG with triple immunostaining of BDNF^+^ (red), LAMP2^+^ (green), and NeuN^+^ (gray) cells. The dashed circles indicate BDNF^+^ neurons. **(N)** Quantification of the number of LAMP2 puncta (green) in BDNF^+^NeuN^+^ cell soma. n = 3 mice per group, *P* < 0.0001. Scale bar = 50 μm (Figure 5F). Scale bar = 5 μm (Figure 5G, 5I and 5M). Data are presented as the mean ± SEM. Two-tailed unpaired *t-*test was used to identify statistically significant differences between datasets (*P < 0.05, **P < 0.01, ***P < 0.001 compared to the control group). n.s., non-significant difference.

Autophagy has an important impact on cell development and survival; hence, we next investigated the expression of autophagy-related proteins, including LC3Ⅱ, SQSTM1/p62, Atg5, and ULK1, in the DG (Figure 5A). Western blot analysis revealed that the expression of LC3Ⅱ (Figure 5B) and Atg5 (Figure 5D) was increased, the expression of SQSTM1/p62 was reduced (Figure 5C), and ULK1 expression did not change (Figure 5E) in CORT mice compared to that in control mice. These results indicated that CORT markedly activated autophagy in DG neurons by increasing Atg5 expression. We also investigated where autophagy occurred in the hippocampal DG. The data showed that autophagy mostly occurred in neurons of the DG (Figure 5F) but was limited in astrocytes (Figure S2A) or microglia (Figure S2B). Similarly, the electron microscopy results showed that compared to those in control mice, the numbers of autophagosomes and autolysosomes in CORT mice were significantly increased (Figure 5G). To verify the above results, we stereotactically injected the DG with an adeno-associated virus expressing a pH-sensitive tandemly tagged LC3 protein. The tandem fusion protein was composed of mCherry, green fluorescent protein (GFP), and LC3 (mCherry-GFP-LC3, Figure 5H), and expression was driven by the neuron-specific promoter hSyn. The results showed that CORT increased the number of autophagosomes (yellow puncta, Figure 5I and 5J) and autolysosomes (red-only puncta, Figure 5I and 5K) in neurons. Accordingly, the ratio of autolysosomes to autophagosomes was also considerably increased in CORT mice compared with that in control mice (Figure 5L).

In neurons, BDNF is synthesized by the Golgi apparatus and endoplasmic reticulum. Hyperactive autophagy in neurons will consume the Golgi apparatus and endoplasmic reticulum, resulting in reduced levels of neuronal BDNF 28, 29. Lysosomal degradation is mainly performed by lysosomes in neurons; therefore, the number of lysosomes can reflect the strength of lysosomal degradation. Using LAMP2 to mark lysosomes, we found that the number of LAMP2-positive puncta in BDNF^+^ neurons was significantly increased in CORT mice compared to that in control mice (Figure 5M and 5N). These results indicated that CORT induces hyperactive autophagy in the hippocampal DG neurons and promotes lysosomal degradation of neuronal BDNF in mice.

### Inhibiting autophagy in DG neurons ameliorates the neuronal reduction in BDNF, rescues AHN, and reverses depression-like behavior induced by CORT in mice

**Figure 6:**
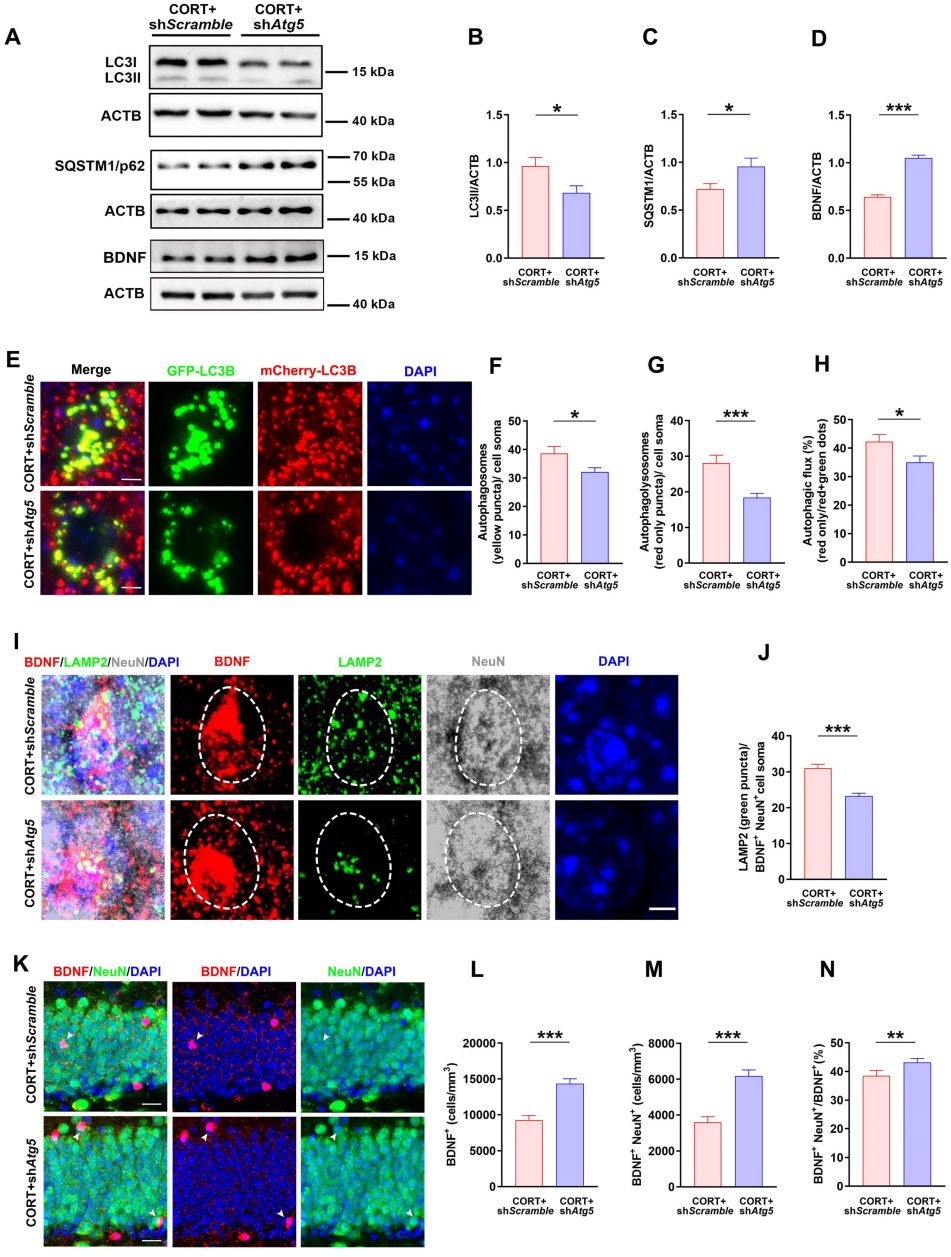
** Inhibiting increases in neuronal autophagy reduces lysosomal degradation of neuronal BDNF and increases neuronal BDNF expression in mice. (A)** Representative western blots analyzing protein expression in the CORT+sh*Scramble* and CORT+sh*Atg5* groups. **(B)** Quantification of LC3II expression. n = 3 mice per group, *P* = 0.0222. **(C)** Quantification of SQSTM1/p62 expression. n= 3 mice per group, *P* = 0.0327. **(D)** Quantification of BDNF expression. n= 3 mice per group, *P* < 0.0001. **(E)** Representative images of AAV-expressed mCherry-GFP-LC3 in the CORT+sh*Scramble* and CORT+sh*Atg5*. **(F)** Quantification of autophagosomes (yellow puncta) in cell soma. n = 4 mice per group, *P* = 0.0482. **(G)** Quantification of autolysosomes (red-only puncta) in cell soma. n = 4 mice per group, *P* = 0.0007. **(H)** Quantification of autophagic flux. n = 4 mice per group, *P* = 0.0336. **(I)** Representative images of DG from CORT+sh*Scramble* and CORT+sh*Atg5* mice with triple immunostaining of BDNF^+^ (red), LAMP2^+^ (green), and NeuN^+^ (gray) cells. Dashed circles indicate BDNF^+^ neurons. **(J)** Quantification of the number of LAMP2 puncta (green) in BDNF^+^NeuN^+^ cell soma. n= 3 mice per group, *P* < 0.0001. **(K)** Representative images of DG in CORT+sh*Scramble* and CORT+sh*Atg5* mice with double immunostaining of BDNF^+^ (red) and NeuN^+^ (green) cells. Arrowheads indicate BDNF^+^NeuN^+^ cells. **(L)** Quantification of BDNF^+^ cells. n = 3 mice per group, *P* < 0.0001. **(M)** Quantification of BDNF^+^NeuN^+^ cells. n = 3 mice per group, *P* < 0.0001. **(N)** Quantification of BDNF^+^NeuN^+^ cells/total BDNF^+^ cells. n = 3 mice per group, *P* = 0.0453. Data are presented as the mean ± SEM. Scale bar = 5 μm (Figure 6E and 6I). Scale bar = 20 μm (Figure 6K). Two-tailed unpaired *t-*test was used to identify statistically significant differences between datasets (*P < 0.05, **P < 0.01, ***P < 0.001 compared to CORT+sh*Scramble* group).

**Figure 7:**
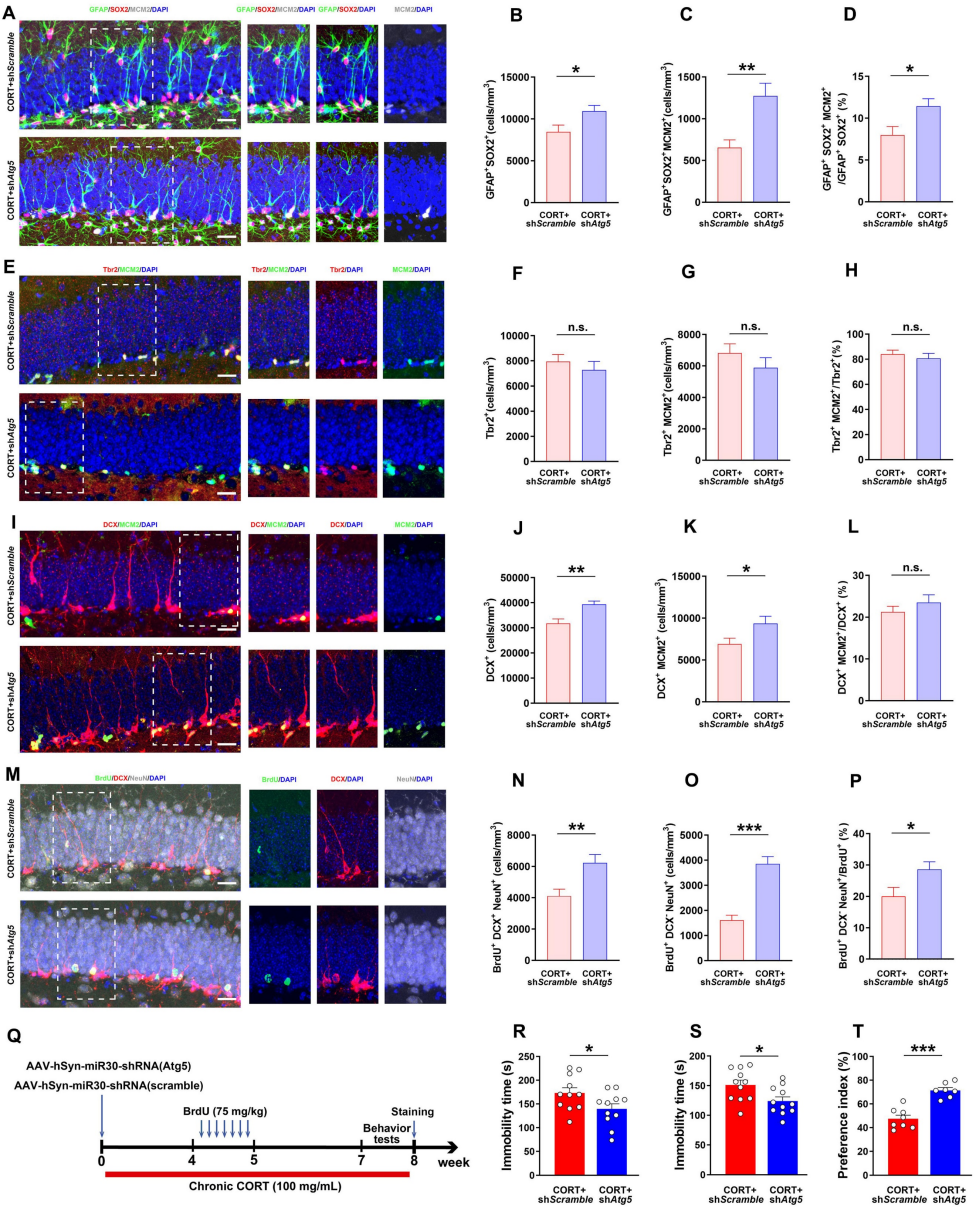
** Inhibiting increased neuronal autophagy rescues AHN and exerts an antidepressant effect. (A)** Representative images of DG in CORT+sh*Scramble* and CORT+sh*Atg5* mice with triple immunostaining of GFAP^+^ (green), SOX2^+^ (red), and MCM2^+^ (gray) cells. **(B)** Quantification of GFAP^+^SOX2^+^ cells. n = 4 mice per group, *P* = 0.0237. **(C)** Quantification of GFAP^+^SOX2^+^MCM2^+^ cells. n = 4 mice per group, *P* = 0.0022. **(D)** Quantification of GFAP^+^SOX2^+^MCM2^+^/GFAP^+^SOX2^+^ cells. n = 4 mice per group, *P* = 0.0135. **(E)** Representative images of DG in CORT+sh*Scramble* and CORT+sh*Atg5* mice with double immunostaining of Tbr2^+^ (red) and MCM2^+^ (green) cells. **(F)** Quantification of Tbr2^+^ cells. n = 3 mice per group, *P* = 0.4581. **(G)** Quantification of Tbr2^+^MCM2^+^ cells. n = 3 mice per group, *P* = 0.2891. **(H)** Quantification of Tbr2^+^MCM2^+^/Tbr2^+^ cells. n = 3 mice per group, *P* = 0.9484. **(I)** Representative images of DG in CORT+sh*Scramble* and CORT+sh*Atg5* mice with double immunostaining of DCX^+^ (red) and MCM2^+^ (green) cells. **(J)** Quantification of DCX^+^ cells. n = 3 mice per group, *P* = 0.0015. **(K)** Quantification of DCX^+^MCM2^+^ cells. n = 3 mice per group, *P* = 0.0352. **(L)** Quantification of DCX^+^MCM2^+^/DCX^+^ cells. n = 3 mice per group, *P* = 0.3326. **(M)** Representative images of DG in CORT+sh*Scramble* and CORT+sh*Atg5* mice with triple immunostaining of BrdU^+^ (green), DCX^+^ (red), and NeuN^+^ (gray) cells. **(N)** Quantification of BrdU^+^DCX^+^NeuN^+^ cells. n = 3 mice per group, *P* = 0.0050. **(O)** Quantification of BrdU^+^DCX^-^NeuN^+^ cells. n = 3 mice per group, *P* < 0.0001. **(P)** Quantification of BrdU^+^DCX^-^NeuN^+^/BrdU^+^ cells. n = 3 mice per group, *P* = 0.0279. Scale bar = 20 μm. **(Q)** Timeline of the experimental procedure to knock down Atg5 via AAV-mediated shRNA expression. **(R)** Immobility time in the TST. n = 11 mice per group, *P* = 0.0414. **(S)** Immobility time in the FST. n = 11 mice per group, *P* = 0.0203. **(T)** Preference index in the exploration stage of the NOR test. CORT+sh*Scramble* (n=8 mice per group), CORT+sh*Atg5* (n=7 mice per group), *P* < 0.0001. Data are presented as the mean ± SEM. Two-tailed unpaired *t-*test was used to identify statistically significant differences between datasets (*P < 0.05, **P < 0.01, ***P < 0.001 compared to CORT+sh*Scramble* group). n.s., non-significant difference.

To explore the effects of hyperactive autophagy in DG neurons on neuronal BDNF, AHN, and depression-like behaviors, we knocked down Atg5 expression in the hippocampal DG neurons of CORT mice using AAV-hSyn-miR30-shRNA (Atg5) (Figure S2C and S2D). The results showed that LC3Ⅱ expression was reduced (Figure 6A and 6B) while SQSTM1/p62 expression was markedly increased (Figure 6A and 6C) in the DG neurons of CORT+sh*Atg5* mice compared to that in the DG neurons of CORT+sh*Scramble* mice. Moreover, the results showed that Atg5 knockdown in the hippocampal DG neurons decreased the number of autophagosomes (yellow puncta, Figure 6E and 6F) and autolysosomes (red-only puncta, Figure 6E and 6G) in neurons. Accordingly, the ratio of autolysosomes to autophagosomes was also considerably decreased in CORT+sh*Atg5* mice than in CORT+sh*Scramble* mice (Figure 6H). This suggests that Atg5 knockdown successfully inhibited CORT-induced autophagy in DG neurons. Moreover, inhibiting hyperactive autophagy in the DG neurons significantly reduced the number of LAMP2 puncta (lysosomes) in BDNF^+^ neurons (Figure 6I and 6J) and increased the expression of BDNF (Figure 6A, 6D and 6L), especially the expression of BDNF in neurons (Figure 6K and 6M). The ratio of BDNF^+^NeuN^+^/BDNF^+^ cells (Figure 6K and 6N) was also increased in CORT+sh*Atg5* mice than in CORT+sh*Scramble* mice. However, differences in the mRNA expression of BDNF gene were not observed between the CORT+sh*Atg5* mice and CORT+sh*Scramble* mice (Figure S2E). Notably, inhibiting hyperactive autophagy in the DG neurons significantly restored the survival and proliferation of NSCs (Figure 7A, 7B and 7C) and neuroblasts (Figure 7I, 7J and 7K), and promoted the survival of newborn immature neurons (Figure 7M and 7N) and newborn mature neurons (Figure 7M and 7O), but had no effects on NPCs (Figure 7E, 7F and 7G). In addition, inhibiting hyperactive autophagy in the DG neurons increased the proportion of proliferating NSCs (Figure 7D) and surviving newborn mature neurons (Figure 7P) but did not affect the proportion of proliferating NPCs (Figure 7H), neuroblasts (Figure 7L), and newborn immature neurons (Figure S2F) in CORT+sh*Atg5* mice. Finally, inhibiting hyperactive autophagy in the DG neurons significantly reduced the duration of immobility in the TST (Figure 7Q and 7R) and FST (Figure 7Q and 7S) and increased the preference index in the NOR test (Figure 7Q and 7T). These data indicate that inhibiting hyperactive autophagy in the DG neurons reverses CORT-induced depressive-like behaviors. Together, these results suggest that knocking down Atg5 expression in the hippocampal DG neurons can rescue the CORT-induced phenotypes of reduced neuronal BDNF expression, abnormal AHN and depressive-like behaviors in mice. These effects might be attributed to inhibition of hyperactive autophagy in DG neurons.

## Discussion

High levels of glucocorticoids are closely associated with depression 30; however, the underlying mechanism remains unclear. In the present study, we used CORT to induce depressive-like behavioral phenotypes and cognitive deficits in mice, which were accompanied by decreased BDNF levels in the DG, especially in neurons. These results are consistent with those of previous studies 5, 6, 10, 11, 13, 19. The hippocampus plays an important role in regulating emotions and cognition 3, and reports have indicated that the clinical effect of antidepressants is closely related to the hippocampus 7, 11. Neurogenesis in the SGZ of the hippocampal DG has attracted extensive attention. Studies have also shown that newborn immature neurons and newborn mature neurons in the DG participate in the regulation of depressive-like behaviors, which may be related to the activation of the neural circuits involving those cells 9, 31. However, to our knowledge, the effect of CORT on AHN in mice, especially on the developmental lineage of NSCs, has not been reported. Our results show that CORT not only depletes NSCs, NPCs, and neuroblasts, but also reduces the proliferation of NSCs, NPCs and neuroblasts, which suggests that CORT impairs the proliferation stage of AHN.

To confirm these results, we used a well-established protocol based on BrdU and Ki67 staining 32 and found that CORT inhibited proliferating cells from exiting the cell cycle. This indicates a negative effect of CORT on the cell cycle kinetics of these cells. Furthermore, CORT significantly reduced the ratio of proliferating NSCs without affecting the ratio of proliferating NPCs and neuroblasts. These results may explain why CORT exposure decreased the number of NPCs and neuroblasts. It has been reported that CORT or stress can reduce the number of NSCs in mice 18, 33, as observed in the present study. In addition, we found that CORT significantly increased NSC apoptosis, which was characterized by an elevated level of cleaved-caspase 3 in NSCs. Caspase 3 is known to regulate cell development and apoptosis 34, and its increase in NSCs suggests that CORT may lead to depletion of the NSC pool by inhibiting the development and promoting the apoptosis of NSCs. In summary, CORT induces aberrant changes in NSCs, which affects the proliferation stage of AHN.

Regarding the differentiation stage of AHN, NSCs may differentiate into immature neurons and finally produce functional mature neurons, which are eventually integrated into the neural circuit 35. Therefore, the number of newborn immature and mature neurons is often used to evaluate AHN. Here, we found that CORT significantly decreased the number of newborn immature and mature neurons, which suggests that CORT impairs the survival of newborn immature and mature neurons. Our findings are consistent with the fact that stress or CORT reduces the production of newborn neurons 3, 12, 27. Notably, the proportion of newborn immature and newborn mature neurons was not affected by CORT, which indicates that CORT had no effect on the differentiation of NSCs. Our results showed that CORT greatly reduced the migration distance of newborn mature neurons without affecting the thickness of the GCL. This suggests that CORT may impair the integration of newborn neurons into neural circuits. Thus, we speculate that during the differentiation stage of AHN, CORT not only impairs the survival of newborn immature and newborn mature neurons, but also reduces the migration of newborn mature neurons, which eventually leads to diminished AHN.

Stress/CORT-induced autophagic death of NSCs has been reported to attenuate AHN and induce cognitive defects 18. However, whether CORT-induced autophagy occurs in DG neurons, or how this autophagy subsequently affects the development and differentiation of NSCs have not been reported. We used the autophagy marker LC3 to co stain different neural cells and found that autophagy occurred mainly in neurons but not in astrocytes or microglia in the DG of CORT mice. Moreover, CORT significantly increased the number of autophagosomes and autolysosomes in neurons and enhanced autophagic flux. Previous studies reported that CORT induced excessive autophagy in primary hippocampal neurons and PC12 cells *in vitro* 36, 37, which was confirmed by our *in vivo* findings. Furthermore, western blot analysis of autophagy-related proteins confirmed that CORT enhanced autophagy in DG neurons, which was validated by both electron microscopy and immunofluorescence. Notably, our results showed that CORT had no effect on the expression of ULK1, an autophagy-related protein, but enhanced the expression of Atg5. Hence, our data indicate that CORT induces hyperactive autophagy in DG neurons at least in part through enhancement of Atg5 expression.

How does neuronal autophagy affect the development of NSCs? As previously mentioned, BDNF is produced by neurons and astrocytes in the brain and plays an important role in the development and survival of NSCs by binding to TrkB receptors on NSCs 38. Our results showed that CORT significantly reduced the protein level and mRNA level of BDNF. Previous studies found that autophagy could selectively degrade mRNA 39, 40. More importantly, both the Golgi apparatus and endoplasmic reticulum, where BDNF is synthesized, are substrates of autolysosomes. Therefore, we speculate that hyperactive neuronal autophagy may degrade the mRNA of BDNF gene or consumes the Golgi apparatus and endoplasmic reticulum, resulting in impaired translation and processing of BDNF and reduced BDNF expression in neurons. To confirm the above hypothesis, we analyzed the lysosomal degradation of BDNF in neurons by using LAMP2, a marker of lysosomes. The results showed that CORT increased LAMP2 expression in BDNF^+^ neurons, which suggests that the hyperactive neuronal autophagy may impair processing of neuronal BDNF. However, it is not clear which mechanism plays a major role in the reduction of neuronal BDNF, or whether both are involved. In general, CORT induces hyperactive autophagy in neurons, which in turn reduces BDNF in neurons, causes abnormal changes in NSCs and AHN, and finally induces depressive-like behaviors.

Next, to further verify the importance of hyperactive neuronal autophagy, we knocked down ATG5 in neurons by RNA interference. This allowed us to investigate the effect of neuronal autophagy on neuronal BDNF, AHN, and depressive-like behaviors. The results showed that the expression of LC3II in CORT+sh*Atg5* mice was greatly reduced compared to that in CORT+sh*Scramble* mice, while the expression of SQSTM1/p62 was substantially increased. This confirms the role of Atg5 in CORT-induced hyperactive autophagy in neurons. Inhibiting hyperactive neuronal autophagy in the DG significantly decreased LAMP2 expression in BDNF^+^ neurons and increased BDNF expression in neurons, but had no effect on the mRNA expression of BDNF gene. These results indicate that inhibiting hyperactive neuronal autophagy may not affect the translation of BDNF but improve the processing of BDNF in neurons, which confirming our hypothesis that neuronal autophagy depleted neuronal BDNF. Moreover, inhibiting hyperactive neuronal autophagy in the DG also significantly reversed the CORT-induced reduction in the number of NSCs, neuroblasts, newborn immature neurons and newborn mature neurons, and increased the proportion of NSCs undergoing proliferation and newborn mature neurons undergoing differentiation. These observations further confirm that autophagy has an important effect on the development of NSCs and AHN 17, 41. Finally, inhibiting hyperactive neuronal autophagy in the DG significantly improved CORT-induced depression-like behaviors and hippocampal-dependent cognitive deficits, including reductions in immobility duration in the TST and FST and increases in the preference index in the NOR test. These results are consistent with those of previous studies that used the stress-induced depression model 20, 21.

In summary, our findings reveal for the first time that CORT acts through a neuronal autophagy-dependent mechanism to reduce the level of BDNF, suppress AHN, and induce depressive-like behavior in mice. These data provide a clue for therapeutic strategies to combat depression by targeting neuronal autophagy. However, several questions remain unanswered. For example, although inhibiting hyperactive neuronal autophagy rescues AHN in CORT mice, it is unclear whether specific molecular mechanisms are involved, such as the glucocorticoid receptor pathway. In the future, we plan to conduct single-cell RNA sequencing of neurons with hyperactive autophagy and identify the key factors/pathways involved in the regulation of NSC development by neurons.

## Supplementary Material

Supplementary figures.

## Abbreviations

AAV: adeno-associated virus
AHN: Adult hippocampal neurogenesis
AP: anterior posterior
Atg5: autophagy-related gene 5
BDNF: Brain-derived neurotrophic factor
BrdU: Bromodeoxyuridine
CORT: corticosterone
DG: dentate gyrus
DV: dorsoventral
EPM: elevated plus maze
FST: forced swimming test
GCL: granular cell layer
LC3: light chain 3
ML: medial lateral
NOR: Novel object recognition
NPCs: neural progenitor cells
NSCs: neural stem cells
OFT: open field test
PBS: phosphate buffer saline
PFA: paraformaldehyde
SGZ: subgranular zone
TST: tail suspension test
