# Supplementary material for: Hyperactive neuronal autophagy depletes BDNF and impairs adult hippocampal neurogenesis in a corticosterone-induced mouse model of depression
Source: Theranostics. 2023 Jan 22;13(3):1059–75. doi: 10.7150/thno.81067 (PMC9925310; doi:10.7150/thno.81067)
Supplement: Supplementary file 1 — Supplementary figures. [file thnov13p1059s1.pdf]

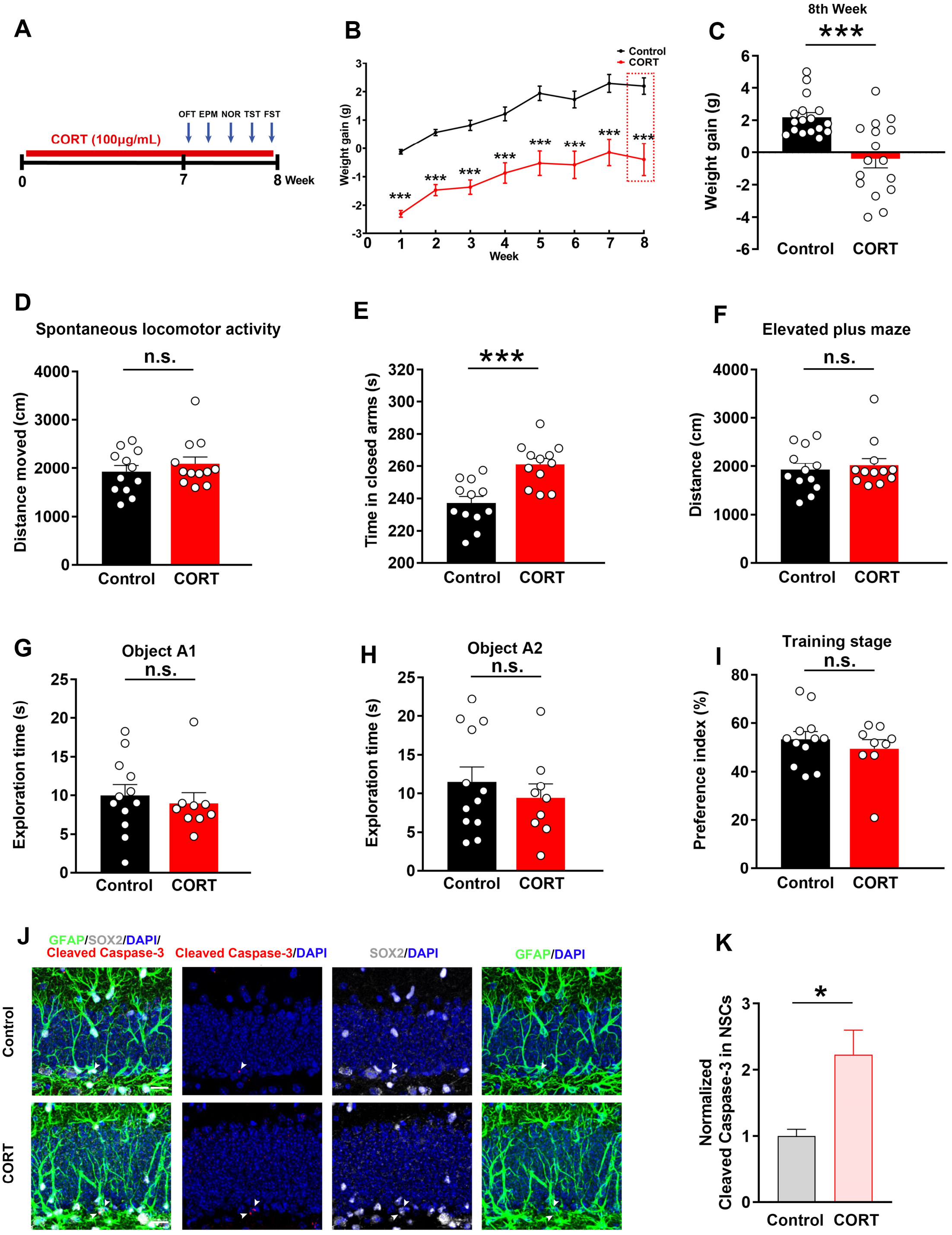

Figure S1. The effect of chronic corticosterone (CORT) treatment on behaviors, NSC apoptosis and neuronal BDNF. (**A**) Timeline of the experimental procedure. (**B**) Line chart showing body weight gain. Control (n = 17), CORT (n = 6), $P < 0.001$. (**C**) Body weight gain in the eighth week. Control (n = 17), CORT (n = 16), $P = 0.0002$. (**D**) Distance

moved while assessing locomotor activity. n = 12 per group, $P = 0.4033$. (**E**) Time spent in the closed arms of the EPM. n = 12 per group, $P = 0.0003$. (**F**) Distance moved in the EPM. n = 12 per group, $P = 0.6474$. (**G**) Exploration time for object A1 in the training stage of the NOR test. n = 12 per group, $P = 0.6176$. (**H**) Exploration time for object A2 in the training stage of the NOR test. n = 12 per group, $P = 0.4432$. (**I**) Preference index in the training stage of the NOR test. Control (n=12), $P = 0.4330$. (**J**) Representative images of control and CORT DG with triple immunostaining of $SOX2^+$ (gray), $GFAP^+$ (green), and cleaved-caspase 3^+ (red) cells. Arrowheads indicate $SOX2^+$/$GFAP^+$/cleaved-caspase 3^+ cells. (**K**) Quantification of $SOX2^+GFAP^+$cleaved-caspase 3^+ cells. n = 4 mice per group, $P = 0.0182$. Scale bar = 20 μm. Data are presented as the mean ± SEM. Two-tailed unpaired *t*-test was used to identify statistically significant differences between datasets ($*P < 0.05$, $**P < 0.01$, $***P < 0.001$ compared to the control group). n.s., non-significant difference.

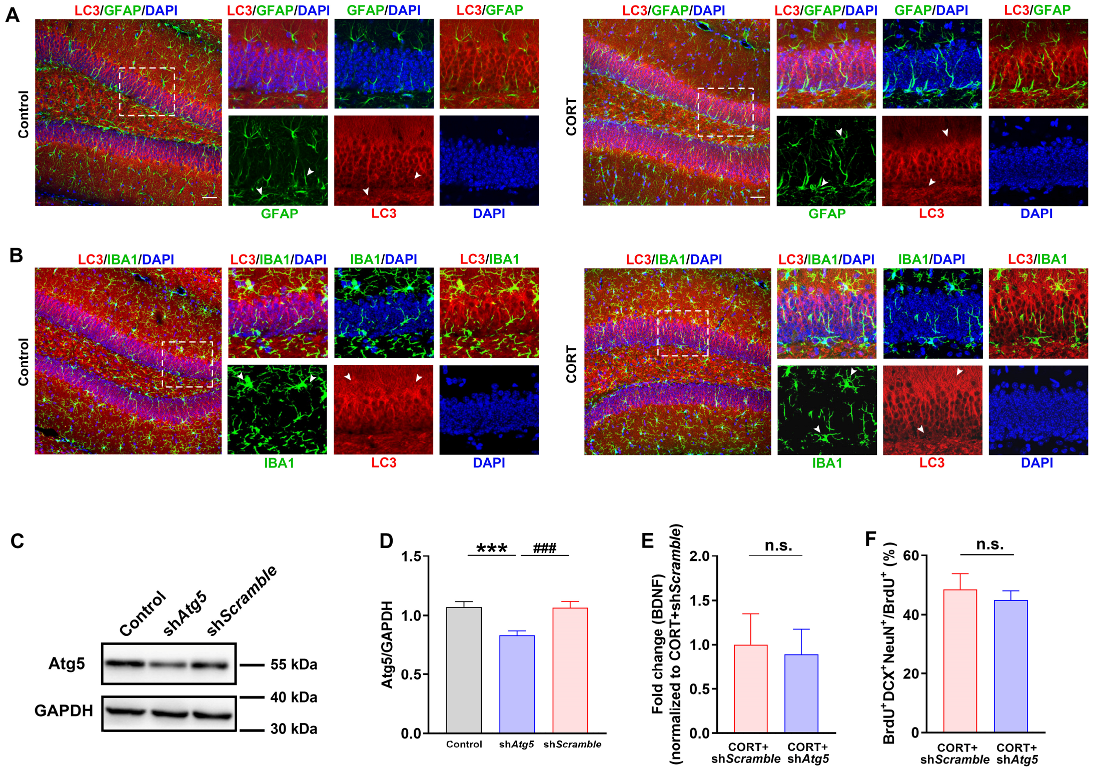

Figure S2. The effect of chronic CORT on autophagy in astrocytes and microglia in mice. (**A**) Representative images of control and CORT DG with double immunostaining of $LC3^+$ (red) and $GFAP^+$ (green) cells. The boxed areas are enlarged in the right panels. White arrowheads indicate $LC3^+/GFAP^+$ cells. (**B**) Representative images of control and CORT DG with double immunostaining of $LC3^+$ (red) and $IBA1^+$ (green) cells. The boxed areas are enlarged in the right panels. White arrowheads indicate $LC3^+/IBA1^+$ cells. (**C**) Representative western blots analyzing protein expression in control, control+sh*Atg5* and control+sh*Scramble* groups. (**D**) Quantification of Atg5 expression. n = 3 mice per group, $P < 0.0001$. (**E**) Quantification of BDNF gene mRNA expression. n = 4 mice per group, $P = 0.8135$. (**F**) Quantification of $BrdU^+DCX^+NeuN^+/BrdU^+$ cells. n = 3 mice per group, $P = 0.5394$. Data are presented as mean ± SEM. The two-tailed unpaired *t*-test was used to identify statistically significant differences between datasets (***$P < 0.001$ compared

to control group, $^{###}P < 0.001$ compared to CORT+sh*Scramble* group). n.s., non-significant difference.
